# Supplementary material for: Imaging and mechanism of DNA–DNA recognition mediated by divalent ions
Source: Nucleic Acids Res. 2026 Aug 25;54(16):gkag817. doi: 10.1093/nar/gkag817 (PMC13504290; doi:10.1093/nar/gkag817)
Supplement: Supplementary file 1 [file gkag817_supplemental_files.zip › Catley_Velasco_SI.docx]

**SUPPLEMENTARY INFORMATION**

**Imaging and mechanism of DNA-DNA recognition mediated by divalent ions**

Thomas E. Catley^1✝^ Victor Velasco-Berrelleza^2,3✝^, Daniel E. Rollins^1^, Alice L. B. Pyne^1^* and Agnes Noy^2,4^*

**Sequence of 339 bp linear minicircle DNA.** The fragments selected for MD simulations are highlighted in colour: cyan for fragment 1, yellow for fragment 2, pink for fragment 3 and green for fragment 4.

^1^TATGTTGTGT ^11^TTTACAGTAT ^21^TATGTAGTCT ^31^GTTTTTTATG ^41^CAAAATCTAA ^51^TTTAATATAT ^61^TGATATTTAT ^71^ATCATTTTAC ^81^GTTTCTCGTT ^91^CAGCTTTTTT ^101^ATACTAACTT ^111^GAGCGAAACG ^121^GGAAGGGTTT ^131^TCACCGATAT ^141^CACCGAAACG ^151^CGCGAGGCAG ^161^CTGTATGGCG ^171^AAATGAAAGA ^181^ACAAACTTTC ^191^TTGTACGCGG ^201^TGGTGAGAGA ^211^GAGAGAGAGA ^221^TACGACTACT ^231^ATCAGCCGGA ^241^AGCCTATGTA ^251^CCGAGTTCCG ^261^ACACTTTCAT ^271^TGAGAAAGAT ^281^GCCTCAGCTC ^291^TGTTACAGGT ^301^CACTAATACC ^311^ATCTAAGTAG ^321^TTGATTCATA ^331^GTGACTGCA

**Supplementary Figures and Tables**

**Figure S1:** The two initial configurations employed to initiate each replica simulation for every pair. The two duplexes were oriented in two distinct azimuthal angles: 0 degrees for replica 1 and 180 degrees for replica 2. They were positioned in parallel, separated by 3.5 nm in accordance with the central DNA axis.

**Figure S2.** Example of time evolution for a simulation presenting highly closed pairing (replica 1 of fragments 2-2 in nickel). (a) Root-mean-squared deviation (RMSd) as a function of time for each cluster of the simulation with respect to their representative structure. (b) Heatmap of the matrices showing distances between axes points across duplexes for the different clusters. Their representative structures are shown below.

**Figure S3.** Example of time evolution for a simulation presenting loose pairing (replica 1 of fragments 4-4 in nickel). **a** Root-mean-squared deviation (RMSd) as a function of time for each cluster of the simulation with respect to their representative structure. **b** Heatmap of the matrices showing distances between axes points across duplexes for the different clusters. Their representative structures are shown below.

**Figure S4.** Condensation in the presence of different divalent ions. Representative images of pairing events in a) 10 mM NiCl_2_, b) 10 mM CaCl_2_, and c) 10 mM MgCl_2_.  Scale bars = 50 nm, height scale = -3 to 4 nm.

**Figure S5.** Quantification of the measured width of DNA from the AFM images. **a)** Process of obtaining the DNA width using a distance transform of the mask generated in TopoStats. Scale bar = 20 nm. **b)** Distribution of the measured DNA widths for all the molecules measured with AFM, with a gaussian fit overlaid with a peak at 6.1 ± 0.4 nm. N = 1220. **c**) Example wide-area AFM image showing a pairing event for 339 bp linear DNA fragments. Scale bar = 100 nm, Height scale = -3 to 4 nm. **d**) Crop of a close pairing event. Scale bar = 50 nm. **e**) Detection of close pairing events based on a pixel-pixel distance threshold of 6 nm.

**Figure S6. (a)** Representative structures for each DNA pair from the replica not shown in Fig. 3, ordered from lowest intermolecular distance (left) to highest (right). Ni^2+^ density maps with an occupancy of 25 times or greater the bulk concentration are shown in cyan. DNA pairings are designated by the fragments that constitute them (1, 2, 3 or 4) together with the replicas 1 and 2 (r1 and r2). **(b)** Representations of planarity corresponding to the paired DNAs above with the front duplex in blue and the rear duplex in red.

**Figure S7.** Intermolecular distances in KCl environments. Inter-helical distances between DNA pairs solvated in 200 mM KCl rarely decreased below 3 nm, confirming the absence of condensation in the presence of monovalent salts. Systems are identified by their constituent fragments (2 and 3) and replicates (r1 and r2), which correspond to the two initial relative orientations of the DNA duplexes. Fragments 2 and 3 were selected for these simulations as they exhibited the highest pairing propensity among fragments sampled from the central and terminal regions of the 339 bp linear DNA, respectively.

**Figure S8.** Deviation from planarity in the simulations presenting parallel pairing. MG labels denote simulations containing magnesium ions, while CA denotes calcium ions. All remaining simulations correspond to nickel ions. Trajectories with 20 mM divalent ions placed at the lowest energetically favourable positions are labelled as LE, while charge-neutralised DNA trajectories with divalent ions placed at the lowest energetically favourable positions are labelled as LEN. The relative deviation from planarity is calculated with respect to the length of the structure formed by the two paired DNA strands.

**Figure S9.** Detailed views of the DNA bridges established by the nickel ions with the longest residence times in the simulations with the lowest intermolecular distance and more planar arrangement (see Table S2). The sequence motifs interacting with Ni^2+^ are highlighted in colour: A is represented in yellow, T in purple, C in orange, and G in green. The GTAC motif participates in all three pairing sites, with the other sequences preserving stable ions located opposite to it. See Movies S1, S3 and S4 of the simulations.

**Figure S10.** Structural outcomes of DNA pairing simulations under Ca^2+^ and Mg^2+^ solvent conditions. Representative structures are shown for all simulations exhibiting physical contact between the two DNA duplexes under Ca^2+^ (top) or Mg^2+^ (bottom) conditions (see Method section for further details). Simulations are identified by the DNA fragments involved (1 or 2) and the corresponding replicas (r1 or r2). Mg^2+^ or Ca^2+^ density maps show an occupancy of 25 times or greater the bulk concentration (in cyan). Insets below paired configurations provide an alternative perspective showing the planarity of the pairing with the front duplex in blue and the rear duplex in red. A comprehensive summary of simulation conditions, ionic strengths, and structural outcomes is provided in Table S1.

**Figure S11.** Time-resolved intermolecular distances for non-pairing DNA trajectories performed under Ca²⁺ or Mg²⁺. Inter-helical distances between DNA duplexes are shown for simulations in Ca²⁺ and Mg²⁺ solvent conditions, including (i) 20 mM of divalent cations randomly distributed (20 mM), (ii) 20 mM of divalent ions placed at the lowest energetically favourable positions (20 mM LE), and (iii) charge-neutralised DNA systems with divalent ions placed at the lowest energetically favourable positions (neutral LE). The traces correspond to simulations that either did not exhibit direct physical contact between the two DNA duplexes or showed only transient contacts that dissociated before the end of the trajectory. Sudden increases in inter-helical distance in some systems arise from the reassignment of one DNA duplex to a neighbouring periodic boundary image. Systems are identified by their constituent fragments (1 and 2) and replicas (r1 and r2). See the Methods section for further details and Table S1 for a comprehensive summary of simulation conditions, ionic strengths, and structural outcomes.

**Figure S12.** Representative structures showing DNA–DNA pairing in 100 mM CaCl_2_ (1–1 r1) (**a**) and 100 mM MgCl_2_ (2–2 r1) (**b**) simulations. Zooms highlight bridging ions (in cyan) with residence times >60%. Sequences hosting ions within the grooves are colour-coded by nucleotide identity: A, yellow; T, purple; C, orange; and G, green. Backbone oxygen atoms involved in ion bridging are shown in red. See Movies S7 and S8 of the simulations and Table S2 for more information regarding bridging ions.

**Table S1.** Summary of molecular dynamics simulations. A total of 40 simulations were performed, each lasting at least 500 ns, resulting in a cumulative duration over 20 µs^a^.

| Salt | Concentration^b^ | Initial ion distribution^b^ | Sequences | Replicas | Pairing^c^ |
| --- | --- | --- | --- | --- | --- |
| NiCl_2_ | 100 mM | Random | 1 – 1 | r1 | Yes |
|  |  |  |  | r2 | Yes |
|  |  |  | 2 – 2 | r1 | Yes |
|  |  |  |  | r2 | Yes |
|  |  |  | 3 – 3 | r1 | Yes |
|  |  |  |  | r2 | Yes |
|  |  |  | 4 – 4 | r1 | Yes |
|  |  |  |  | r2 | Yes |
|  |  |  | 1 – 2 | r1 | Yes |
|  |  |  |  | r2 | Ends |
|  |  |  | 1 – 4 | r1 | Yes |
|  |  |  |  | r2 | Yes |
|  |  |  | 2 – 3 | r1 | Yes |
|  |  |  |  | r2 | Yes |
|  |  |  | 2 – 4 | r1 | Yes |
|  |  |  |  | r2 | Yes |
| KCl | 200 mM | Random | 2 – 2 | r1 | No contact |
|  |  |  |  | r2 | No contact |
|  |  |  | 3 – 3 | r2 | No contact |
|  |  |  | 2 – 3 | r2 | No contact |
| MgCl_2_ | 100 mM | Random | 1 – 1 | r1 | Yes |
|  |  |  |  | r2 | Ends |
|  |  |  | 2 – 2 | r1 | Yes |
|  |  |  |  | r2 | Yes |
| Mg^2+^ + KCl | 10 mM (24 Mg^2+^) + 150 mM | Random | 2 – 2 | r1 | No contact |
|  |  |  |  | r2 | No contact |
|  | 10 mM (24 Mg^2+^) + 150 mM | Low-energy + Random | 2 – 2 | r1 | No contact |
|  |  |  |  | r2 | Ends |
|  | Neutral (64 Mg^2+^) + 150 mM | Low-energy + Random | 2 – 2 | r1 | No contact |
|  |  |  |  | r2 | Yes |
| CaCl_2_ | 100 mM | Random | 1 – 1 | r1 | Yes |
|  |  |  |  | r2 | Ends |
|  |  |  | 2 – 2 | r1 | Yes |
|  |  |  |  | r2 | Ends |
| Ca^2+^ + KCl | 10 mM (24 Ca^2+^) + 150 mM | Random | 1 – 1 | r1 | Cross |
|  |  |  |  | r2 | No contact |
|  | 10 mM (24 Ca^2+^) + 150 mM | Low-energy + Random | 1 – 1 | r1 | Yes |
|  |  |  |  | r2 | Ends |
|  | Neutral (64 Ca^2+^) + 150 mM | Low-energy + Random | 1 – 1 | r1 | Ends |
|  |  |  |  | r2 | Yes |

^a^Please refer to the method section for more details regarding system preparation.

^b^Ions were distributed randomly in most of the simulations, except for some simulations run in physiological salt conditions (i.e combining low concentrations of Mg^2+^/Ca^2+^ and 100 mM KCl) where they were placed in the most energetically favourable positions. In parenthesis the number of divalent ions added in the solvation box.

^c^Some simulations resulted in non-parallel orientations, for example involving end contacts (‘Ends’) or crossed configurations (‘Cross’). Only simulations presenting parallel pairing were included in measurements of inter-helical distance and groove alignment.

**Table S2**. DNA atoms and sequences interacting with bridging ions (residence times >60%) in selected trajectories showing optimal pairing across various divalent cation neutralization conditions^a^. To avoid end effects, ions that interacted with the terminal 5 bp were excluded.

| Fragments paired | Ion | Residence time (%) | DNA sequence^b^ | DNA atoms^c^ |
| --- | --- | --- | --- | --- |
| Nickel | | | | |
| 2-2 r1 | 1^st^ | 89 | 5'G**TA**C3' | N3/O2 (mg) – OP1/OP2 (bb) |
|  | 2^nd^ | 79 | 5'A**AG**A3' | N3/O2 (mg) – OP1/OP2 (bb) |
| 2-2 r2 | 1^st^ | 74 | 5'G**TA**C3' | N3/N3 (mg) – OP1/OP2 (bb) |
|  | 2^nd^ | 69 | 5'G**TA**C3' | N3/O2 (mg) – OP1/OP2 (bb) |
|  | 3^rd^ | 63 | - | OP1 (bb) - OP1 (bb) |
| 2-3 r1 | 1^st^ | 90 | 5'G**TA**C3' | N3/O2 (mg) – OP1/OP2 (bb) |
|  | 2^nd^ | 84 | 5'C**TA**C3' | N3/O2 (mg) – OP1/OP2 (bb) |
| 2-4 r2 | 1^st^ | 96 | 5'G**T**AC3' | N3 (mg) – OP1/OP2 (bb) |
|  | 2^nd^ | 96 | 5'T**TA**A3' | N3/O2 (mg) – OP1/OP2 (bb) |
|  | 3^rd^ | 93 | 5'A**CC**G3' | N7/O6 (Mg) – OP1/OP2 (bb) |
| Calcium | | | | |
| 1-1 r1   100mM | 1^st^ | 97 | 5'G**AG**G3' | N3/O2 (mg) – OP1/OP2 (bb) |
|  | 2^nd^ | 94 | 5'A**TG**G3' | N3/O2 (mg) – OP1/OP2 (bb) |
|  | 3^rd^ | 91 | - | OP1/OP2 (bb) – OP1/OP2 (bb) |
|  | 4^th^ | 88 | - | OP1/OP2 (bb) – OP1/OP2 (bb) |
|  | 5^th^ | 86 | 5'C**GC**G3' | O6/N7 (Mg) – OP1/OP2 (bb) |
|  | 6^th^ | 69 | - | OP1/OP2 (bb) – OP1/OP2 (bb) |
|  | 7^th^ | 66 | 5'A**GG**C3' | N3/O2 (mg) – OP1/OP2 (bb) |
|  | 8^th^ | 65 | - | OP1/OP2 (bb) – OP1/OP2 (bb) |
|  | 9^th^ | 65 | - | OP1/OP2 (bb) – OP1/OP2 (bb) |
|  | 10^th^ | 60 | 5'C**A**G3' | N7 (Mg) – OP1/OP2 (bb) |
| 1-1 r2 neutral LE | 1^st^ | 96 | 5'G**CA**G3' | N3/N3 (mg) – O1P/O2P (bb) |
|  | 2^nd^ | 90 | 5’C**A**G3’ | N3 (mg) – O1P (bb) |
|  | 3^rd^ | 74 | 5'G**G**C3' | N2 (mg) – OP2 (bb) |
|  | 4^th^ | 71 | - | O1P/O2P (bb) – O1P/O2P (bb) |
|  | 5^th^ | 67 | - | O1P/O2P (bb) – O1P/O2P (bb) |
| Magnesium | | | | |
| 2-2 r1  100 mM | 1^st^ | 100 | 5'A**CC**G3' | N2/N3 (mg) – O1P/O2P (bb) |
|  | 2^nd^ | 100 | - | O1P (bb) – O1P/O2P (bb) |
|  | 3^rd^ | 100 | - | O1P/O2P (bb) – O1P/O2P (bb) |
|  | 4^th^ | 100 | - | O1P/O2P (bb) – O1P (bb) |
|  | 5^th^ | 100 | - | O1P/O2P (bb) – O1P/O2P (bb) |
|  | 6^th^ | 93 | - | O1P/O2P (bb) – O1P/O2P (bb) |
|  | 7^th^ | 72 | 5'A**CC**G3' | O6/N7 (Mg) – O1P (bb) |
| 2- 2 r2 neutral LE | 1^st^ | 100 | - | O1P/O2P (bb) – O1P/O3' (bb) |
|  | 2^nd^ | 100 | - | O2P/O2P (bb) – O1P/O2P (bb) |
|  | 3^rd^ | 93 | 5’T**A**C3’ | N3/O3’ (mg/bb) – O2P (bb) |
|  | 4^th^ | 92 | 5'C**AC**C3' | N6/O6 (Mg) – O1P/O2P (bb) |
|  | 5^th^ | 78 | 5'A**AG**A3' | N7/O6 (Mg) – O1P/O2P (bb) |
|  | 6^th^ | 73 | - | O1P/O2P (bb) – O2P (bb) |

^a^ neutral LE refer to charge-neutralised DNA simulations with divalent ions placed at the lowest energetically favourable positions.

^b^ Sequence is only highlight when the ion interacts with atoms from the nucleobases.

^b^ Atoms in the minor groove are marked as 'mg', in the major groove as ‘Mg’, backbone atoms as 'bb', and phosphate oxygens as OP1 and OP2.

**Supplementary Movies**

**Movie S1.** The first replica of the simulations of fragment 2, paired with itself and solvated in Ni^2+^, showing the bridging ions with residence times over 60% in red and the remaining ions in cyan.

**Movie S2.** The second replica of the simulations of fragment 2, paired with itself and solvated in Ni^2+^, using the same colour code as in Movie S1. Both replicas are selected among the best simulations showing DNA-DNA condensation in Ni^2+^, as they exhibit similar levels of alignment and planarity (Fig. 3 and S6). Non-planar structures typically involve the DNA ends in the condensation, resulting from the limited length of our modelled DNAs (Fig. S4).

**Movie S3.** The first replica of the simulations of fragment 2 paired with fragment 3 and solvated in Ni^2+^, using the same colour code as in Movie S1. This replica is selected to illustrate the best alignment between the two fragments, as the resulting pairing structure exhibits a notably greater planarity compared to the second replica (Fig. 3, S4 and S6).

**Movie S4.** The second replica of the simulations of fragment 2 paired with fragment 4 and solvated in Ni^2+^, using the same colour code as in Movie S1. This replica is selected to illustrate the best alignment between the two fragments, as the resulting pairing structure exhibits a notably greater planarity compared to the first replica (Fig. 3, S4 and S6).

**Movie S5.** The second replica of the simulations of fragment 1, paired with itself, and solvated under low Ca^2+^ concentrations. DNA charges are neutralized by Ca^2+^ ions placed at the lowest-energy favourable positions, supplemented by 150 mM KCl. The colour scheme matches Movie S1, with K^+^ ions shown in white. This simulation is selected to illustrate the best alignment in Ca^2+^, as it exhibits the highest levels of alignment (Fig. 5).

**Movie S6.** The second replica of the simulations of fragment 2, paired with itself, and solvated under low Mg^2+^ concentrations. DNA charges are neutralized by Mg^2+^ ions placed at the lowest-energy favourable positions, supplemented by 150 mM KCl. The colour scheme matches Movie S1, with K^+^ ions shown in white. This simulation is selected to illustrate the best alignment in Mg^2+^, as it exhibits the highest levels of alignment (Fig. 5).

**Movie S7.** The first replica of the simulations of fragment 1, paired with itself, and solvated in 100 mM CaCl_2_, using the same colour code as in Movie S1.

**Movie S8.** The first replica of the simulations of fragment 2, paired with itself, and solvated in 100 mM MgCl_2_, using the same colour code as in Movie S1.
